# A Gesture Elicitation Study of Nose-Based Gestures

**DOI:** 10.3390/s20247118

**Published:** 2020-12-11

**Authors:** Jorge-Luis Pérez-Medina, Santiago Villarreal, Jean Vanderdonckt

**Affiliations:** 1 Intelligent and Interactive Systems Lab (*SI*^2^ Lab), Universidad de Las Américas, Quito 170504, Ecuador; 2 LouRIM Institute, Université Catholique de Louvain, B-1348 Louvain-la-Neuve, Belgium; santiago.villarreal@uclouvain.be (S.V.); jean.vanderdonckt@uclouvain.be (J.V.)

**Keywords:** agreement rate and score, gesture elicitation study, gestural interaction, wearable sensor

## Abstract

Presently, miniaturized sensors can be embedded in any small-size wearable to recognize movements on some parts of the human body. For example, an electrooculography-based sensor in smart glasses recognizes finger movements on the nose. To explore the interaction capabilities, this paper conducts a gesture elicitation study as a between-subjects experiment involving one group of 12 females and one group of 12 males, expressing their preferred nose-based gestures on 19 Internet-of-Things tasks. Based on classification criteria, the 912 elicited gestures are clustered into 53 unique gestures resulting in 23 categories, to form a taxonomy and a consensus set of 38 final gestures, providing researchers and practitioners with a larger base with six design guidelines. To test whether the measurement method impacts these results, the agreement scores and rates, computed for determining the most agreed gestures upon participants, are compared with the Condorcet and the de Borda count methods to observe that the results remain consistent, sometimes with a slightly different order. To test whether the results are sensitive to gender, inferential statistics suggest that no significant difference exists between males and females for agreement scores and rates.

## 1. Introduction

Sensors have become so miniaturized that they can be integrated into virtually any wearable device or everyday object, such as smart watches and glasses, thus offering new forms of interaction [1]: a sensor is able to recognize human movements performed on some dedicated parts of the human body [2,3,4].

One primary form of gestural interaction has recently been linked to a new source of input with myography [5]: electrooculography (EOG) sensing [6]. An electrooculogram allows the measurement of the corneo-retinal standing potential difference between the front and the back of the human eye. Electrodes are placed by pair “left/right of the eye” or “above/below the eye”. When the eye moves from its central position toward one electrode, it senses the positive side of the retina while the other electrode perceives the negative side of the retina. A movement is obtained by calculating the change in position for a short duration of time.

**Figure 1:**
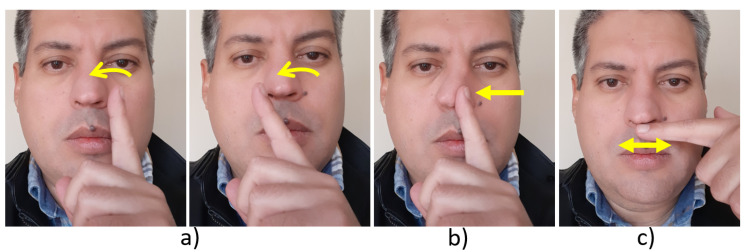
Examples of the ItchyNose [6,7] gestures: Flick (**a**), Push (**b**), and Rub (**c**).

The Itchy Nose [6] is another representative example. It embeds, in the bridge of a pair of eyeglasses, an electrooculography-based sensor. The J!NS MEME, is a wearable computer solution that detects eye and head movements. The Itchy Nose recognizes five nose-based gestures [7]: left/right flick (Figure 1a), left/right push (Figure 1b), and rub (Figure 1c). Beyond these five system-defined gestures, the vocabulary of nose-based gestures can be largely expanded by user-defined gestures [8], which has not been done so far. Since the Itchy Nose is the first system of its kind, more experimental implementations can be expected in the future.

Nose-based gestures are original as they offer unique opportunities: they are intended to be discreet, being performed without attracting the attention of the user in public [6,7], they replace gestures when all other parts of the human body are either occupied or covered, such as in cold circumstances [9] or when no device is imposed for touchless interaction [10], they serve for authentication [11], they convey emotional states [9], they operate for nose-pointing [12]. Overall, these kinds of gestures are envisioned for two interaction families:

1. Dual task interaction: a primary task is ongoing (e.g., a conversation during a meeting) and a secondary task (e.g., a phone call) occurs, potentially interrupting the primary one, and requires some discreet interaction to minimize interference with the primary task. For example, a Rubbing gesture discreetly ignores a phone call without disturbing the conversation too much. This is inspired by the dual task performance, a test for assessing the cognitive workload in psychology [13]
2. Eyes-free and/or touch-free interaction [14]: a task should be carried out by interacting with a system without requiring any visual attention and physical touch. Gestures are discreetly performed on the face, an always-accessible area in principle.

This paper aims at addressing a main research question: what is the vocabulary of nose-based gestures preferred by end users for executing various actions? This main question includes two sub-questions: (1) Does the method used to measure preference impact the results or, in other words, would the vocabulary of user-defined gestures change if another measurement is performed? (2) Are these results sensitive to gender or, in other words, would male and female participants prefer nose-based gestures differently or consistently since their respective preference is subject to social acceptance? To address these questions, this paper makes the following contributions:

1. A gesture elicitation study conducted with two groups of participants, one composed of 12 females and another one with 12 males, to determine their user-defined, preferred nose-based gestures, as detected by a sensor [7], for executing Internet-of-Things (IoT) actions.
2. Based on criteria for classifying the elicited gestures, a taxonomy of gestures and a consensus set of final gestures are formed based on agreement scores and rates computed for all actions.
3. A set of design guidelines which provide researchers and practitioners with some guidance on how to design a user interface exploiting nose-based gestures.
4. A comparison of the results obtained by agreement scores and rates with respect to those obtained with two other measurement methods, i.e., the Condorcet [15] and the de Borda [16] methods.
5. An inferential statistical analysis testing the gender effect on preferred gestures.

To this end, this paper is organized as follows: Section 2 discusses the work related to nose-based interaction and gesture elicitation studies. Section 3 details the research method used in the experiment conducted. Section 4 discusses the results of this experiment by classification criteria, by agreement score and rate, and by gender. Section 6 compares the results obtained in Section 4 for preferred gestures with those obtained by two other measurement methods. Section 5 suggests a set of six design guidelines. Section 7 concludes the paper and presents some avenues to this work.

## 2. Related Work

This section defines some terms for facial anatomy, reviews prior works on nose-based interaction are presented, and summarizes the outcome of a GES.

**Facial Anatomy.** The external appearance of the nose is made up of a surface and a series of skeletal structures [17]: the root is the part located between the eyebrows, the root is connected to the rest of the nose by means of a bridge. The tip of the nose is known as the apex, the ala is a cartilaginous structure that covers the lateral side of each naris, or nostril opening, one on each side of the apex. The nostrils are surrounded by the nasal septum and the cartilaginous wings of nose. The philtrum consists of a concave surface. This connects the apex to the upper tip, a very protruding and stable point [18], and the dorsum nasi is the length of the nose.

**Nose-based Interaction.** Nose-based interaction appears in 1991 as a joke, it was introduced by using the pointed nose on a surface to move objects [19]. Two decades later, this hoax becomes a reality, something that was never thought by its authors. In reality, NoseDialSee https://buhmann-marketing.de/nosedial/index.html is an iOS application enabling end users to dial contacts by pointing the nose on contact targets. The software allows end users to customize the format, size and position of the contacts according to the nose anatomy. This application, unique in its style, is useful in contexts of use where there is no other pointing mechanism available, i.e., in extreme hand-busy conditions. For example, a user could avoid removing gloves to dial a person in a freezing environment. Swiping left/right scrolls among contacts, holding the nose 0.2 sec dials a contact, and double tapping returns to the initial application that was suspended after a call was answered. Similarly, the Finger-Nose stylus See https://variationsonnormal.com/2011/04/28/finger-nose-stylus-for-touchscreens/. is a funny prototypical device replacing finger touch-based gestures by nose-based gestures on a surface.

The review of the literature allows us to observe that the social acceptability of facial gestures has been the object of studies. Rico and Brewster [20] investigated the social acceptability of nose-based gestures. They found that some facial gestures are more perceived in public than others. Freeman et al. [21] reports that most preferred regions are: cheek (34%), forehead (16%), jaw (8%), apex of the nose and others like chin, ear, and temple having more or less 7%. Although the cheek received the largest percentage (34%) due to its maximal surface, the alae and philtrum were not tested, thus leaving the potential of the nose full surface unexplored. SNOUT [22] conducted a non-standard study of touch interaction using mobile devices. The study had 13 participants who performed various nose interactions. Subsequently, these interactions were compared. As a result of the survey carried out, the authors found five design guidelines for nose-based interaction. NoseTapping [9], allows end users to tap or swipe a touchscreen with their nose. With this application, a great need to use these input modalities in contextual situations was revealed. For instance, restricted user contexts, short use cases. Likewise, its impossibility was revealed in more complex functions, such as writing a message or editing content. Nose-based gestures remain an underexplored area in the field of on-skin gesture interaction, like in SkinWatch [23], Serendipity [24], and SensIR [25].

**Gesture Elicitation Studies.** Capturing, analyzing, and understanding end-user needs, preferences and behavior in relation to the new interactive technology from the initial stages of a design process allows the work team to have valuable information to design the characteristics of a more effective and efficient product. This process is known as guessability studies [26] or Gesture Elicitation Studies (GES) [8] to understand users’ preferences for gesture input in a wide variety of user contexts [27]. For example, Wobbrock et al. [8] revealed users’ preferences for multi-touch gestures in a context based on interactive tabletops. Vatavu [28] addressed mid-air gestures to control a smart television. Ruiz et al. [29] investigated users’ preferences for motion gestures by using smartphones. The outcome of a GES characterizes users’ gestural behavior with valuable information for practitioners, such as designers and developers, as well as for end users regarding the consensus level between participants, the most frequent gesture proposals for executing a given action with a particular device, and insights into users’ mental models.

GES have been primarily conducted along the three dimensions of the context of use [27]: users and their interactive tasks [30], their platforms, devices, and associated sensors [8,31], and the environments in which they are working [32]. Since their inception, GES initially focused on some particular platform, device, or sensor, ranging from the most popular and widespread ones to the most recent and original ones: tabletops [8,31], mobile interaction [29], smart television [33], virtual hologram [34], and radar-based sensors [10]. GES then focused on gestures performed on particular physical component, such as the trackpad [35] or on the bezels of a smartphone [36]. The advent of cross-platform interaction resulted in GES for Multi-Display Environments (MDE) [37] and between any platform combination, such as for migrating contents across mobile phones, public displays, and tabletops [38].

Villarreal et al. [39] reported that GES were performed on almost all human limbs: the most frequent cover hand gestures [40] and their fingers [2,41], on-skin freehand gestures [42], arms gestures [43], head gestures[44,45], while the least frequent investigate limbs with less mobility, such as the mouth [33], the head and shoulders [46], the torso [47], and the belly [48]. GES can be the object of study in contexts where it is required to understand any particular physical capacity or human ability or the deficiency thereof. It turns out that the existence and the frequency of GES is correlated with the mobility level of the studied limb.

In conclusion, this paper motivates a GES for exploring nose-based gestures: this region has never been subject to any GES [39] although it is extensively used for face recognition since the tip of the nose is the most prominent and stable part of the human face [18], the gesture vocabulary is unknown, apart from the Flick, Rub and Tap gestures [7], no analysis, either qualitative or quantitative, has been reported in the literature, about the gestures preferred by users when exploring nose-based gestures. Studying nose-based gestures and natural expressions represents a great challenge. Most of the available video corpus and gesture sets do not have one or more factors that are crucial for our analysis. These datasets and corpora did not capture any nose interactions. Seipp & Verbert [49] identified “null” gestures as having no particular meaning, but that could be recognized and mapped onto a command. Among them are: Rub chin, Tap finger on face, Scratch head. The nose is common to all human. Therefore, male and female should in principle elicit the same gestures, except if these people experience social acceptance differently [20]. Whether the gender influences the gestures elicited remains untouched, knowing that human face is subject to social acceptation before preference [21]. Inspiration could also come from the field of communication, where body language expresses a wide range of knowledge about the use and interpretation of gestures [50]. Morris [50] conducted a study of 20 gestures in 40 European countries. As a result, the author found that the meanings of nasal puncture vary, yet they all share the metaphor of “sniffing out problems”.

## 3. Experiment

A Gesture Elicitation Study (GES) following as reference a well-known methodology was carried out. The initial methodology is defined in [8,26,41,51,52] to collect users’ preferences for nose gestures. Kendon [53] defines a gesture as any particular type of body movement performed in any amount of dimensions (e.g., linear to spatial), which falls into two categories: involuntary movements and actions, which can be practical actions (e.g., a manipulation gesture) or gestural actions (gesticulation and autonomous gestures). To distinguish a nose gesture from others, a *nose gesture* is defined as any movement involving the nose as an intentional movement of the nose itself (called *nose movement*, e.g., holding the nostrils open, moving the bridge) or any hand, respectively device, movement on the nose (*hand-to-nose gesture*, e.g., pushing, flicking, and rubbing the apex, swiping the dorsum nasi, respectively *device-to-nose gesture*, e.g., nose-pointing [9]). Any combination or repetition of these movements is a compound gesture.

### 3.1. Participants

Twenty-four people took part in the experiment voluntarily (12 Males, 12 Females; aged from 12 to 68 years, M=30.2, SD=12.2). The participants were recruited for the experiment through a list of contacts in various organizations. The recruitment process was carried out following the convenience sampling guidelines to participate in the experiment [54].

All the participants declared to be right-handed. Their occupations of the participants included director, teacher, psychologist, secretary, employee, retirees, and students in domains such as transportation, nutrition, law, history, chemistry, and economics. Usage frequencies were captured for various devices, in which they are: smartphones, tablets, computers, game console and depth cameras such as Kinect. All participants reported that they make frequent use of smartphones and computers in daily life.

The age groups are distributed as follows: 2 people below 18 years (8%), 8 people between 18 years and 24 (33%), 9 people between 25 and 34 (38%), 4 people between 45 and 54 (17%), and only one person above 55 years (4%). The age group was chosen for our participants to be as representative as possible for adopters of wearable technology. The percentage of individuals who use wearable is the highest for the age group 25–34 years old (30.8%), followed by the 18–25 y group (29.1%), and the 35–44 y. group (25.3%). All participants reported unaware of the existence and use of the nose interaction. All participants reported previously unaware of the existence and use of the nose interaction.

### 3.2. Apparatus

The experiment took place in a usability laboratory to guarantee complete control over all stages of the experiment. A computer screen was provided to the participants so that they could visualize the referents used by the experiment. All the gestures made by the participants were recorded by a camera. The camera allowed to capture the faces of the participants, in this way the region of the nose could be covered as well as their hands. To keep the study focused on the topic, the participants were asked to limit their movements to their hands and fingers without any other instrumentation.

### 3.3. Procedure

The procedure consists of three sequential phases conducted individually for each participant.

#### 3.3.1. Pre-Test Phase

Prior to the experimentation phase, the participants were welcomed by the researchers. They were then asked to sign an informed consent document, compatible with the GDPR regulation. Then they were given detailed information about the study, the experimentation setting, and the entire experiment process. They were also invited to complete a sociodemographic questionnaire followed by a creativity test and a motor-skill test.

The researchers collected the sociodemographic data (e.g., age, gender, handedness) about each participant to use some of these parameters in the study. The questionnaire also asked a series of questions about the use of technologies. All questions in the questionnaire were based on a seven-point Likert scale [55] ranging from 1 = strongly disagree to 7 = strongly agree. The participants’ creativity was studied through http://www.testmycreativity.com/: the instrument consists of a series of questions that allowed us to obtain the levels of creativity of each participant. Finally, the Motor-skill test, described in [56], was applied to check the participants’ dexterity.

#### 3.3.2. Test Phase

In this phase, the participants were informed about the meaning and use of the nose interaction. Each participant had the opportunity to ask questions about their concerns. The participants were informed about the following tasks that they had to perform. The researchers also reported on the types of gestures allowed, consistent with our definition. The participants worked bearing in mind that no restriction was imposed on them, neither technological nor on the recognition of gestures. This had allowed to preserve the natural and intuitive character of elicitation.

Each session implemented the original GES defined in [8]. The participants were presented with a series of *referents*. They consisted of actions to control various objects in IoT. From them, the participants made two gestures to execute those references. The condition was to perform those gestures that fit well with the referents, apart from being easy to produce and above all to remember. Participants were instructed to remain as natural at all times as possible. The referents are assigned randomly. Each participant received a list of random numbers generated with the link: www.random.org.

The *thinking time* was timed in seconds. This time allowed to capture the duration between the first sample of the referent and the moment in which the participant knew what gesture she would make. Each gesture produced by the participant was valued between a range of 1 to 10. The evaluation allowed the participant to express how appropriate the gesture was for the referent. Each session took approximately 45 min per participant. The number of experimenters present in the experiment was 3. Only one of the participants had the responsibility of presenting the referent from the random list of numbers. The remaining experimenters supported the logistics of the experimental process at all times.

#### 3.3.3. Post-Test Phase

The participants’ sessions culminated with an invitation to answer the IBM Post-study System Usability Questionnaire (IBM PSSUQ) [57]. This questionnaire allows participants to express their level of satisfaction with the usability of the scenario and the testing process. This instrument was used because it is effective and empirically validated, its effectiveness has been demonstrated with large numbers of participants bearing in mind a significant set of stimuli [58]. The IBM PSSUQ is widely applicable to any interactive system, its reliability coefficient is α=0.89 in relation to its results and the appraisals of perceived usability of the system [57]. The questions in the IBM PSSUQ questionnaire are measured using a 7-point Likert scale, a value of 1 represents a strongly disagree while a value of 7 shows a strongly agree appreciation. In the IBM PSSUQ four measures are computed: items from 1 to 5 correspond to the system usefulness (SysUse), the quality of the information or (InfoQual) is represented by items from 6 to 11, quality of the interaction or (InterQual) consists of the items from 12 to 15, and finally the system quality or (Overall) is represented by the item 16.

### 3.4. Design

Our study was within-subjects with two groups (female vs. male) and with one independent variable: Referent, a nominal variable with 10 conditions, representing common actions executed in IoT: Turn On the TV, Turn Off the TV, Start Player, Turn the Volume up, Turn the volume down, Go to the next item in a list, Go to the previous item in a list, Turn Air Conditioning On, Turn Air Conditioning Off, Turn Lights On, Turn Lights Off, Brighten Light, Dim Light, Turn Heat On, Turn Heat Off, Turn Alarm On, Turn Alarm Off, Answer a phone call, End Phone Call.

### 3.5. Quantitative and Qualitative Measures

Five measures were captured to understand the preferences as well as the performance of the participants nose gestures:

1. Agreement Scores—“A(r)” [8] and Co-agreement Rates—“AR(r)” [51] were obtained for each Referent “*r*” condition by using the equation:
  (1) $$A\left(r\right)=\sum_{P_{i}\subseteq P}\;{\left(\frac{\left|P_{i}\right|}{\left|P\right|}\right)}^{2}\geq AR\left(r\right)=\frac{\left|P\right|}{|P|-1}\sum_{P_{i}\subseteq P}\;{\left(\frac{\left|P_{i}\right|}{\left|P\right|}\right)}^{2}-\frac{1}{|P|-1}$$
  where *r* means the referent for which a gesture is elicited, |P| refers to the number of elicited gestures, and $|P_{i}|$ means the number of gestures for the i-th which is subgroup of *P*.
2. Participants’ Creativity was evaluated using an online creativity instrument. The test returns a result between the values 0 and 100 where higher scores denote more creativity. The results are calculated from a set of responses grouped into categories: (1) abstraction of concepts from the presentation of ideas; (2) connection between things/elements or objects without an apparent link; (3) perspective shift in terms of space, time, and other people; (4) curiosity to change and improve things/elements and situations accepted as the norm; (5) boldness to push boundaries beyond the normally accepted conventions; (6) paradox the ability to accept and work with concepts that are contradictory; (7) complexity the ability to operate with a large amount of information; and (8) persistence to derive stronger solutions even when good ones exist.
3. Participants’ fine motor skills was measured with a standard motor test of the NEPSY (a developmental NEuroPSYchological assessment) test batteries [56]. The test consists of touching each fingertip with the thumb of the same hand for eight times in a row. Higher motor skills are reflected in less time to perform this task.
4. Thinking-Time measures the time, in seconds, elapsed to elicit any gesture for a referent.
5. Goodness-of-Fit represents participants’ subjective assessment, as a rating between 1 and 10, of their confidence about how well the proposed gestures fit the referents. Participants could elicit their two gestures in any order with a different Goodness-of-Fit.

## 4. Results and Discussion

A total amount of nine hundred 12 (912) gestures were elicited from 2 groups × 12 participants × 19 referents × 2 gestures. The groups were formed bearing in mind the following criteria

1. Dimension: the cardinality of the gesture space: 0D (point), 1D (line), 2D (plane), 3D (space).
2. Laterality: which side(s) have been used to issue the gesture, unilateral (when a gesture is elicited only on one side of the dorsum nasi) or central (if the gesture is issued on the edge).
3. Gesture motion: which is the intensity of the movement stroke (as a snap or a hit), static (if performed on a single location) or dynamic (if the speed or movement is changing over time).
4. Nature: describes the meaning of a gesture with four values adapted from [8]: symbolic gestures depict commonly accepted symbols conveying information, such as emblems and cultural gestures, e.g., the Call me gesture performed with the thumb and little finger stretched out, or swiping the index finger from left to right; metaphorical gestures give shape to an idea or concept, such as using the thumb to press a button on an imaginary remote control to turn on/off the TV set; abstract gestures have no symbolic or metaphorical connections to their referents; physical gestures refer to the real world physics.
5. Number of fingers: how many fingers were involved.
6. Finger type: type of finger involved in the elicited gesture.
7. Path type: direct, flexible, without any particular path.
8. Movement axis: stationary, horizontal, vertical, or composed.
9. Area: above the nose, under the nose, left part of the dorsum nasi, right part, center, multiple areas.

### 4.1. Gesture Classification

**Figure 2:**
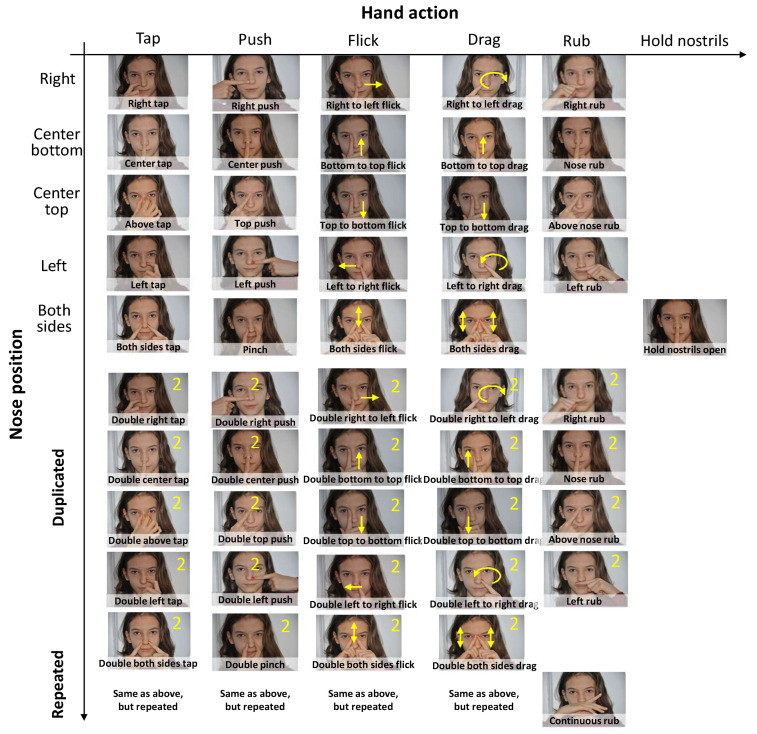
Taxonomy of nose-based gestures: by hand action, nose position, and factor of repetition.

The 912 elicited gestures are classified into 23 categories, with a sub-category when relevant to finely distinguish the nose area involved in the gesture, thus producing 53 individual gestures. The sub-categories below are defined so that they can be used consistently throughout the categories (Figure 2). For instance, *0.5, respectively *0.6 sub-categories, indicate that the gesture was issued on the right part of the dorsum nasi, resp. the left part:

1. Tap: tap any side of the dorsum nasi with the back or the top of one or several fingers with one hand (1.0), on the center (1.1), with two hands on both sides of the nose (1.2), repeated center tap (1.3), right tap (1.5), left tap (1.6).
2. Double tap: tap two times on the center (2.1), both sides of the nose (2.2), right (2.5), left (2.6), above the nose (2.7).
3. Triple tap: tap three times in a row on the center (3.1), right (3.5), left (3.6), above (3.7).
4. Flicking: from right to left (4.5), from left to right (4.6), from the top of the dorsum nasi to the bottom (4.7).
5. Pushing: center push (5.1), right push (5.5), left push (5.6).
6. Rubbing: rub once on right/left side (6.5/6.6), above the nose (6.7), continuous rub on the right/left (8.8/8.9).
7. Double rubbing: repeat rubbing two times in a row.
8. Triple rubbing: repeat rubbing three times in a row.
9. Drag: stays pressed from the initial point to the final one with the right (9.1) or left hand (9.2), from bottom to top (9.3) or vice versa (9.4), from right to left (9.5) or inverse (9.6).
10. Double drag: on the right (10.5), on the left (10.6), from bottom to top (10.7), from top to bottom (10.8).
11. Triple drag: drag repeated three times in a row.
12. Quadruple drag: drag repeated four times in a row.
13. Pinch: when two fingers come far from each other.
14. Double pinch: when two fingers come far, close to each other.
15. Circle: draw a circle on a facet.
16. Double flicking: rapid unilinear movement repeated twice.
17. Hold nostrils open: as defined.
18. Push nose up: push on the nose with a finger up
19. Wrinkle: pulling up the nose without hands.
20. Double wrinkle: repeat the wrinkle two times.
21. Pull on nose: pull the nose with a finger.
22. Finger in nose: in the right/left nostril (22.5/22.6).
23. Sniffing: right part (23.5), left part (23.6).

Some nose gestures have never been elicited, because of their underlying connotation or social acceptance [21]. For example, the Snook gesture where the thumb is put on the apex and the rest of the hand is extended in space to mean defiance, disrespect, or derision; the waggling gesture is avoided for the same reason; the Wiggle nose, which moves a nostril up and down or left to right, is physically uncomfortable to produce; the slapping gesture because it could be painful, it communicates forgetfulness [50]; and gestures with a strong connotation: (a) putting the full hand on the nose, (b) rubbing the whole hand, (c) the “shut up” gesture, and (d) the Smell gesture. On the other hand, some gestures were not discarded although initially it was thought that they would be, such as the rub gesture, a sign of deception or nervousness [50].

### 4.2. Agreement Scores and Co-Agreement Rates

**Figure 3:**
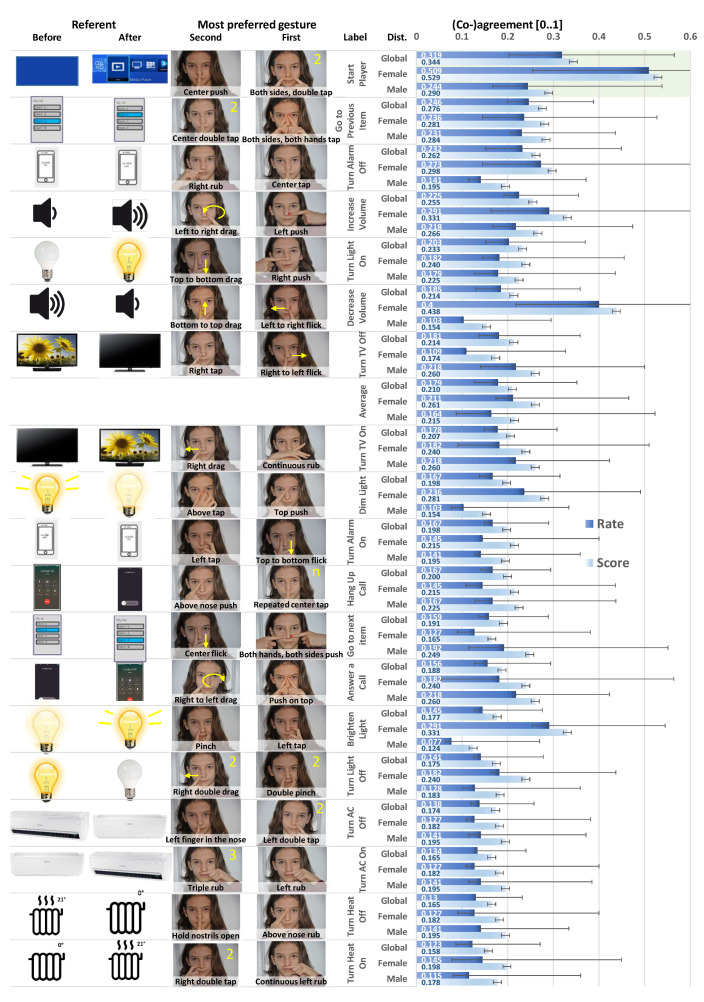
Co-agreement rates [51] agreement scores [8] by gender (global, male, and female) with the two most preferred gestures by referent, sorted in decreasing order of their co-agreement rate. Error bars show 95% confidence intervals (α=0.05) for the rates and standard errors for the scores.

Figure 3 shows the agreement scores (bottom) and co-agreement rates (top) obtained for each Referent conditions sorted in decreasing order of the co-agreement rate. The values are decomposed into the female group (purple), the male group (blue), and the global sampling (green). For each referent, the first and the second most frequently elicited gestures, classified according to the above list, are reported. The ordering of agreement scores and co-agreement rates remains consistent from one computation to another, except for two pairs of referents (depicted by red arrows): Dim light was ranked higher according to its rate than for its score and Hang up call was ranked lower according to its rate than for its score and comes just a little bit before Dim light. The same phenomenon occurs with the pair of Turn AC On and Turn Heat Off referents.

Categories of the first most frequently elicited gestures are: Tap (7), Push (5), Flick (3), Rub (3), Pinch (1). Categories of the second most frequently elicited gestures are: Tap (4), Drag (6), Rub (3), Push (2), Flick (1), Pinch (1), Finger on nose (1), and Hold nostrils open (1). The Tap gesture is the most preferred for both ranks. Although Push and Flick often occur for the first choice, participants tend to rely on other types of gestures for their second choice, like Drag, Finger in the nose and Hold nostrils open, which never appears as a first choice. Rub is common to both categories with a medium frequency.

Overall, agreement rates are small in magnitude, between 0.123 and 0.319 for the global sampling (M=0.179, SD=0.048), between 0.109 and 0.509 for the female group (M=0.211, SD=0.104), and between 0.77 and 0.244 for the male group (M=0.164, SD=0.049). Regarding the female group, 17/19=89% rates belong to the medium range and 2/19=11% rates are high according to Vatavu and Wobbrock’s method [51] to interpret the magnitudes of agreement rates. It turns out that most of these rates are superior to those given for the male group: 1/19=5% belongs to low value, 18/19=95% belong to medium value. These results are very similar to the other rates reported in the GES literature ([51], (p. 1332)) that summarizes agreement rates of 18 studies, for which the smallest value (0.108) was reached by Liang et al. [59] and Seyed et al. [37] for motion+surface and multi-display gestures, respectively. According to the recommendations [51], our results fall inside medium consensus (<0.3) category.

Beyond agreement scores and rates, it was also necessary to know whether female and male would elicit different gestures for the same set of referents, which can be expressed as:

$H_{0}=$ both groups of female and male have equal agreement rates,

$H_{1}=$ there is a difference among the agreement rates of the k=2 groups.

Vatavu and Wobbrock [51] introduced a statistical test for comparing agreement rates of k≥2 independent groups and a measure to compute agreement shared between these independent groups. Each individual agreement rate captures how much consensus there is within its female or male but, considered alone, cannot describe the consensus between groups.

Therefore the freely accessible AGATe [51] software was used to compute $C_{Rb}$, the co-agreement rates between our two independent groups and $V_{bg}$, the variation in agreement for repeated-design experiments. Out of the 19 referents, only two cases were identified with a low *p*-value. For the Turn down volume, the following results are obtained: $V_{bg}\left(2,n=24\right)=144.500$, p=0.009, Male: AR=0.091, CI 95%=[0.091,0.288], Female: AR=0.348, CI95%=[0.227,0.697], $C_{Rb}$(Male, Female) =0.153, a value for which the post-hoc tests gave: $V_{bg}\left(2,n=24\right)=144.500$, p=0.009. For the Turn alarm off: $V_{bg}\left(2,n=24\right)=60.500,p=0.064$, Male: AR=0.167, CI 95%=[0.152,0.409], Female: AR=0.333,CI95%=[0.182,0.697], $C_{Rb}$ (Male, Female) =0.215, a value for which the post-hoc tests gave: $V_{bg}\left(2,n=24\right)=60.500$, p=0.064. AGAte returned non-significant results with two exceptions: 0.161 for Answer phone call and 0.217 for Brighten lights.

### 4.3. Further Analysis and Gender Effect

#### 4.3.1. Gender

**Figure 4:**
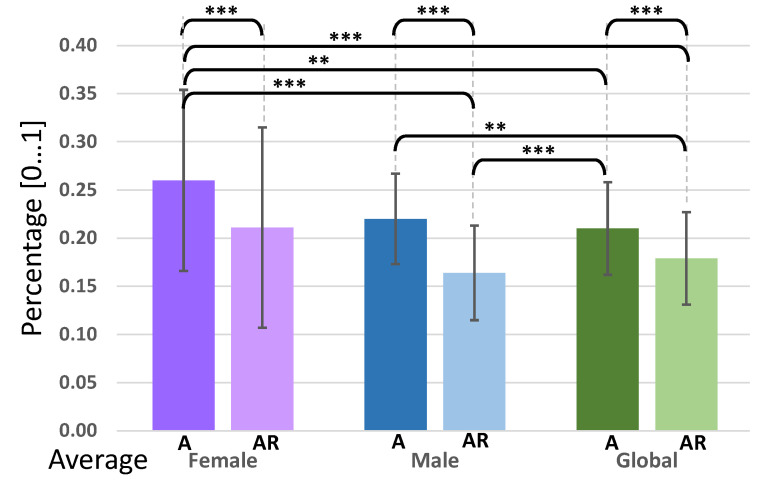
Agreement scores (A) and co-agreement rates (AR) by gender.

An independent-samples *t*-test was conducted to compare agreement scores and agreement rates conditions. First of all, when examining the groups respectively (Figure 4), there was a highly significant difference in agreement for scores and rates conditions within the female group (A: M=0.260, SD=0.094, AR: M=0.211, SD=0.104; $t_{\left(18\right)}=-15.696$, $p\;\leq \;0.001{\;}^{***}$), within the male group (A: M=0.220, SD=0.047, AR: M=0.0164, SD=0.049; $t_{\left(18\right)}=-39.174$, $p\;<\;0.001{\;}^{***}$), and for the global sampling (A: M=0.210, SD=0.048, AR: M=0.179, SD=0.048; $t_{\left(18\right)}=-51.994$, $p\;\leq \;0.001{\;}^{***}$). These results are aligned with Equation (1) stating that AR(r)≤A(r). There was a significant difference ($t_{\left(18\right)}=-3.639$, $p\leq 0.01{\;}^{**}$) in the values for the male score (M=0.220, SD=0.047) and the global rate (M=0.179, SD=0.048). There was also a highly significant difference ($t_{\left(18\right)}=-4.653$, $p\;\leq \;0.001{\;}^{***}$) in the values for the male rate (M=0.164, SD=0.049) and the global score (M=0.210, SD=0.048). At first glance, female agreement values seem to be higher than for male and for the global sampling (which would suggest that female come to a better agreement than male): the respective averages for female are always higher than their male and global counterparts, but their standard deviations are also the widest with respect to male and global. After a closer look, there was a significant difference only in some very specific cases. The only significant difference ($t_{\left(18\right)}=-4.116$, $p\;\leq \;0.001{\;}^{***}$) found between female and male was found in the values for the female score and the male rate. Since these two metrics vary only by two corrective terms, this may suggest that the correction is welcome. With respect to the global sampling, there was a significant difference ($t_{\left(18\right)}=-3.212$, $p\;\leq \;0.01{\;}^{**}$) in the values for the female score (M=0.260, SD=0.094) and the global score, as well as a highly significant one ($t_{\left(18\right)}=-5.286$, $p\;\leq \;0.001{\;}^{***}$) for the female score and the global rate. All others seven t-tests out of the 15 conducted did not reveal any significant difference. No significant difference was found between female vs. male agreement scores and rates ($t_{\left(18\right)}=1.799$, p>0.05, *n.s.*).

#### 4.3.2. Type and Dimension

**Figure 5:**
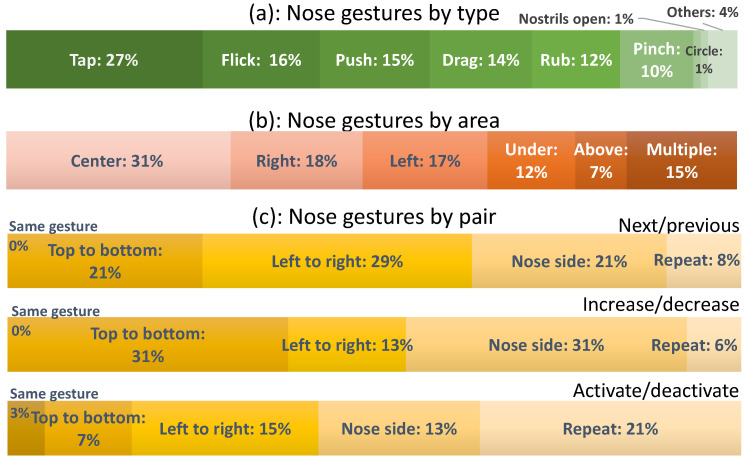
Distribution: (**a**) by type, (**b**) by area, (**c**) by pairs.

Figure 5a depicts how gestures are distributed by type. All gestures elicited less than 5 times (<1% of the sampling) fall in the Others category (4% in total). The most frequently elicited gesture is the Tap, a 0D gesture involving only one point, with one third (27%) of the total sampling. Five other gestures similarly represent one tenth of the total sampling: Flick (2D-16%), Push (0D-15%), Drag (1D-14%), Rub (1D-12%), and Pinch (0D-10%). The Circle (1%) was the only 2D symbol gesture and Hold nostrils, the only 3D gesture. The preference goes to the gestures with less dimensions: 0D (52%), 1D (26%), 2D (17%), and 3D (5%).

#### 4.3.3. Area

Figure 5b depicts how gestures are physically distributed over the areas of the human face: participants largely prefer centered gestures (31%) because they do not need to distinguish laterality. Left (17%) and right (18%) faces are considered equal when chosen. Under the nose was selected in 12% of cases, and above in 7% of cases. Although single areas represent a total amount of 85%, multiple areas were selected in 15% of cases. These results refine suitable areas [21].

#### 4.3.4. Pairs of Commands

The set of referents actually contains nine pairs of semantically related referents, such as opposite, complementary or mutually exclusive. Activate/deactivate pairs cover two-state actions: turn TV/AC/light/ heat/alarm on/off, and Answer/end phone call. Increase/decrease pairs cover a range of values: Increase/decrease volume, Next/previous, Brighten/dim lights, pair only covers: Go to next/previous item in a list. Since referents were presented randomly, participants sometimes complained that they did not remember the gesture they elicited for a previous referent linked to the current one.

Figure 5c depicts which reasoning has been used by participants for these various pairs of referents. Participants hope to observe some logic and/or some reasoning when they elicited gestures coming in pairs. However, it was observed that 30% of elicited gestures did not follow any such logic or reasoning. For Activate/deactivate pairs, Repeat was the most frequent pattern (21%), followed by changing the face of the dorsum nasi (13%), gesture direction (15% left/right and 7% top/bottom). Only one gesture category was used for the same pair (3%). To address the question of which variables influence participants to elicit gestures without any apparent logic or reasoning (see Table 1), some test was performed, but no such correlation was found with creativity (Pearson’s ρ=0.117), with age (Person’s ρ=0.065), with familiarity of devices (Pearson’s ρ=−0.321), and thinking time (Pearson’s ρ=0.215) (n=24). After checking Levene’s test for equality of variances and t-test for equality of means, an independent-samples t-test was conducted to compare creativity, age, items, familiarity with devices, and thinking time. Only one correlation was found between creativity and familiarity with devices (ρ=0.410 significant with α=0.05: 2-tailed) among all possible combinations (See Table 2 and Table 3).

#### 4.3.5. User Satisfaction with Nose Interaction

**Figure 6:**
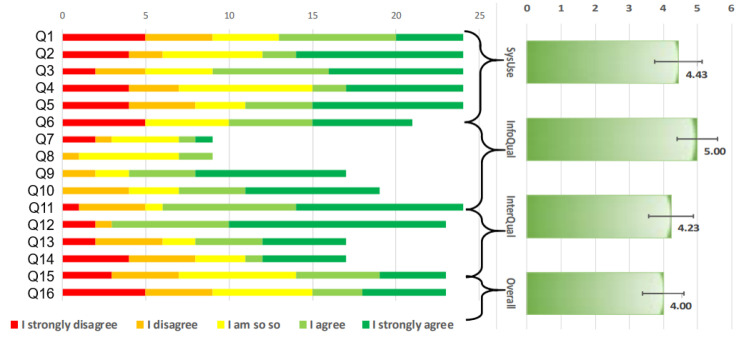
Results of the IBM PSSUQ.

Figure 6 reports the results from the IBM PSSUQ questionnaire. The results express the subjective satisfaction of the participants with respect to the interaction of the nose according to the experiment presented in this document. Error bars allow observing a confidence interval of 95%. The four measures of the PSSUQ questionnaire are considered valid to support the correlation with perceived usability, as long as their value is greater than or equal to 5 on a scale of ranges from 1 to 7.

Participants were very reliable in their answers to this questionnaire (Guttman’ λ−2 gives a score of 0.9701, which is usually wished for high-stakes decisions). Only Information quality (InfoQual: M=5.00, SD=1.48) reaches this threshold with a wide standard deviation for this type of measure, though. System usefulness (SysUse: M=4.43, SD=1.72), Interaction quality (InterQual: M=4.23, SD=1.65), and Overall satisfaction (Overall: M=4.00, SD=1.50) all share a value below 5, which suggests that participants were not quite subjectively satisfied with nose interaction. Two reasons may explain this: the nose is an area that is sensible to suitability in public spaces (the body language is quite related to some gestures [50]).

It was found that the nose gestures are not easy to reproduce, but it is even difficult to perform them consistently and above all, It was found that there is no guidance, there are no immediate user comments on how the gestures should be emitted. Also, there are no immediate comments on how they could be recognized and then trigger an action. Some of the participants stated that they were divided between two aspects: (1) the desire for some guidance or feedback and (2) the guarantee that only the resulting action that is being executed should be the only feedback due to the discretion. These statements are also partially contrasted with the individual questions reported by the participants. The group of questions contained in the category InfoQual were considered “not appropriate” by the participants, consequently, fewer values are reported in Figure 6). However, the reality related to the quality of the information is that this group of questions are considered positive because of the discretion goal. It was also observed that all the remaining questions corresponding to the other measures of the instrument received disagreeing ratings. However, these appreciations never exceeded 5/24=20%) of participants. Finally, it was observed that the participants expressed a satisfactory trend towards efficiency in carrying out the tasks, where questions Q3 and Q6 obtained the highest results.

#### 4.3.6. Nose-Based Gesture Recognition

This section discusses some mechanisms useful for recognizing nose-based gestures as we devised them. The context of use, which is made up of the end-user and the tasks, the device(s), and the environment, of course determines appropriate mechanisms to recognize nose-based gestures.

The gesture capture and recognition process with Itchy Nose is ensured by a sensing technique for detecting finger movements on the nose. Itchy Nose is based on electrooculography (EOG) sensing and on-body interaction [60]. Three EOG sensors embedded in the J!NS Meme are located around the nose [60]: two on the nose pads and one on the nose bridge. Five EOG signals with a probability (i.e., Left/right Push, Left/right Swipe, and Rub) captured by these sensors are sent via Bluetooth to a remote computer where the signals are processedAn open-source GUI gesture toolkit for J!NS Meme is available at https://github.com/sebaram/jins-gesture-toolkit. and the gestures are subsequently classified by the Random Decision Forest method [61]. This configuration is intended to recognize nose-based gestures only. Thus, gestures based on the smart glasses themselves, such as spectacle-based gestures or holding glasses, were not tested although they could be recognized by the sensors as they are close to the sensors. Face-based gestures, such as cheek touching or pushing, tongue moving, cannot be recognized with this method since they are located too far away or in an inaccessible area.

When the end-user does not possess smart glasses, computer vision offers multiple methods for automatic analysis of facial actions [62]. In [63], several face detection and recognition methods were evaluated in order to provide a complete image-based face detection and recognition with high recognition rate, even from the initial step, such as in video surveillance. Methods are proposed based on performed tests on face databases with variations in terms of subjects, poses, emotions, races, and light conditions. Despite these studies, self- expression of these facial expressions is not sufficient to recognize nose-based gestures: finger movement recognition [64] should be involved. Facial expression recognition combined with finger movement recognition should enable us to recognize more nose-based gestures being inspired by our taxonomy (Figure 2).

Facial expression and finger movement recognition methods for nose-based gestures will have their primary resource the use of an effective face detection [65,66] and hands recognition [67]. Consequently, techniques largely vary depending on their source input (e.g., still images, pictures, videos, and real-time streams) and their scientific approach. Support Vector Machines (SVMs) [68], k-Nearest Neighbor (k-NN) [69,70] and other classification methods could solve this classification problem.

## 5. Design Guidelines

Based on our results on preferred gestures, some design guidelines were devised:

1. Match the gesture dimension to task dimension. Used referents cover 0D and 1D tasks. Participants prefer gestures whose dimension is consistent with the task dimension, such as tap for activate/deactivate, tap to select, swipe to scroll. pinch and reverse pinch to shrink or enlarge an object. There is no need to add any extra dimension to the task dimension.
2. Prefer gestures with low dimension. From all elicited gestures, the amount of preferred gestures dramatically decreases with their dimension to the point that probably only 0D and 1D gestures are required as the minimum. Higher dimension gestures were always coming afterwards.
3. Prefer larger areas over small ones. Larger areas (e.g., the dorsum nasi) are adequate for 1D gestures such as scrolling, swiping gestures while small areas (e.g., the ala, the apex or the philtrum) are available for 0D gestures.
4. Favor repetition as a pattern over location. When a gesture is repeated, the repetition factor replaces the fine-grained distinction between individual gestures belonging to the same category. Participants tend to rely less frequently on the physical areas, such as changing the face of the dorsum nasi or preferring the apex.
5. Favor centrality instead of laterality. Gestures that are independent of any laterality are easier to produce and remember than asymmetric ones. For instance, swiping on the dorsum nasi is easier than on any face.
6. Use location only as a last factor. Location could distinguish between gestures, but only as the last refining factor.

## 6. Other Measures for Elicited Gestures

Agreement captures whether some consensus emerges from participants who elicited gestures for the same referent. An agreement measure is computed globally for each referent and is based on their frequency of selection, as reported in Figure 3. To adopt a complementary view on agreements and to identify any particular variation in the agreements, two other methods for consolidating votes from participants on candidates were performed: the Condorcet method [15] and the de Borda method [16], which is used when the first one is unable to identify a *Condorcet winner*. Each method ranks candidates (here, rank elicited gestures) for a selection based on votes (here, based on elicitation and goodness-of-fit). To choose a winner, the Condorcet method is based on the rule: for each referent, select the elicited gesture (if one exists) that beats each other elicited gesture in exhaustive pairwise comparison. The de Borda method is based on the rule: for each referent, select the elicited gesture that on average stands highest in the participants’ rankings. To rank the elicited gestures, Condorcet’s rule is: rank the elicited gestures in descending order of their number of victories in exhaustive pairwise comparison with all the other gestures. Borda’s rule is: rank the elicited gestures in descending order of their standing in the participants’ rankings. In this method, it is common to give the lowest score to the last preferred candidate and to increase the score with the ranking: a score of 1, resp. 2, is assigned to for the second, resp. first, most frequently elicited gesture. Condorcet’s and de Borda’s winners are two methods that must choose an elicited gesture with a claim to democratic legitimacy. The Goodness-of-Fit could introduce an additional weight to moderate the confidence with which participants elicited gestures. This explains why two gestures were captured per participant and per referent.

The de Borda method with weights based on Goodness-of-Fit returns the following list: Both side double tap, Both sides-both hands, Center tap, Center push, Right tap, Left push, Left to right drag, Right push, Top to bottom drag, Left to right flick, Repeated center tap, Right rub, Continuous rubbing, Right drag, Top push, Pinch, Above tap, Top to bottom flick, Double pinch, One hand tap, Above nose push, Center flick, Left tap, Two hands push, Right double drag, Wrinkle, Left double tap, Left finger, Left rubbing, Triple rubbing, Circle, Hold nostrils open, Right double tap. More or less, this list confirms most of the initially selected gestures, but in a different order of preference, apart for the most preferred gestures such as Tap, Push, Drag, and Flick. Some gesture appeared in a more favorable position, such as pinch. Some gestures disappeared, such as some individual rubbing, probably replaced by other gestures which were considered insignificant up to now, like wrinkle and circle. The circle was the only real 2D gesture operated and considered to be the simplest drawing possible that could be produced consistently. The de Borda method without weight, but with a score 1 or 2 and defined above, give more or less a similar list with little variations.

## 7. Conclusions and Future Work

A gesture elicitation study with two samples of equal size (12 females and 12 males) was conducted. The study elicited a series of 912 nose-based gestures (a few nose movements and a large set of hand-to-nose gestures) for 19 referents associated with frequent IoT tasks. These initially elicited gestures are then classified according to several criteria to come up with a classification of 53 individual (unique) gestures falling into 23 categories of gestures, each category potentially having sub-categories. The final consensus set consists of 38 gestures for nose-based interaction (Figure 3 gives the two most frequently elicited gestures per referent). Beyond classification, no significant difference between female and male in the gestures elicited was found. The only significant difference was found between the female score and the corresponding global score. However, no significant difference was found between male and female for the same agreement score and rate. There were some significant differences, though, across the measures (e.g., between female scores and male rates). The analysis confirmed that there is indeed always a significant difference between agreement scores and rates, the last being always inferior or equal to scores. Based on the analysis of the elicited gestures, some design guidelines are suggested for designing nose-based gestures, which could be applicable to wearable devices, sensors, and mobile applications.

Due to the discussion on agreement scores and rates, Scores were also calculated with the Condorcet and the de Borda methods, with a weight based on the goodness-of-fit provided by each participant or without. This analysis suggests that the resulting consensus set remains more or less constant in its selection, but that the order of most preferred gestures within the set could change, with local variations. For instance, two pairs of gestures are swapped when comparing agreement scores and rates (Figure 3). Although participants were asked to express their overall preference through the goodness-of-fit, they were not asked to provide a separate score for social acceptance, like in [21]. From the informal comments gathered during the session, participants also reported that they would never elicit some gestures for different reasons, such as those expressing negative feelings of the body language [50]. This may suggest that a future elicitation study should incorporate not only the most preferred gestures, but also discard the most unwanted gestures. Some participants reported in this case of interaction that they could accommodate different nose gestures taken from the consensus set, but they absolutely want to avoid producing unwanted gestures. Therefore, it is possible to discard the least preferred gestures with a negative filter while keeping the most preferred ones with a positive filter.

Automatic gesture identification for nose interactions represents one of the next steps in our research. The results found from the gesture elicitation study presented in this document will provide a valuable input to start the experiments.

## Figures and Tables

**Table 1:** Descriptive statistics of variables by genre.

Variable	Gender	Mean	Standard Deviation	Standard Error M.
Creativity	Female	64.503	7.780	2.345
	Male	57.825	10.274	2.849
Unlogical items	Female	3.36	2.157	0.650
	Male	2.08	1.977	0.548
Device familiarity	Female	4.000	0.721	0.217
	Male	3.754	1.042	0.289
Thinking time	Female	7.406	4.825	1.455
	Male	6.891	3.118	0.864
Age	Female	30.82	9.888	2.981
	Male	29.69	14.620	4.055

**Table 2:** Correlations between variables. (*) represents the correlation value between creativity and familiarity with devices among all possible combinations.

		Creativity	Age	Unlogic Items	Familiarity	Think Time
Creativity	Pearson c.	1	0.080	0.117	0.410 *	0.071
	Sig. (2-tld.)		0.712	0.587	0.047	0.742
Age	Pearson c.	0.080	1	0.065	−0.307	−0.259
	Sig. (2-tld.)	0.712		0.761	0.144	0.222
Unlogic items	Pearson c.	0.117	0.065	1	−0.321	−0.215
	Sig. (2-tld.)	0.587	0.761		0.126	0.313
Familiarity	Pearson c.	0.410 *	−0.307	−0.321	1	−0.302
	Sig. (2-tld.)	0.047	0.144	0.126		0.151
Thinking time	Pearson c.	0.071	0.259	0.215	−0.302	1
	Sig. (2-tld.)	0.742	0.222	0.313	0.151	

**Table 3:** Independent Samples Test.

		Levene’s Test for Equality of Variances		*t*-Test for Equality of Means						
		F	Sig.	t	df	Sig. (2-Tailed)	Mean Difference	Std. Error Difference	95% Confidence Interval of the Difference	
									Lower	Upper
Creativity	Equal variances assumed	0.577	0.456	1.767	22	0.091	6.67825	3.77911	−1.15914	14.51564
	Equal variances not assumed			1.809	21.775	0.084	6.67825	3.69096	−0.98092	14.33742
Unlogic items	Equal variances assumed	0.534	0.473	1.524	22	0.142	1.287	0.844	−0.465	3.038
	Equal variances not assumed			1.512	20.597	0.146	1.287	0.851	−0.485	3.058
Familiarity device	Equal variances assumed	0.207	0.654	0.660	22	0.516	0.2462	0.3732	−0.5277	1.0200
	Equal variances not assumed			0.680	21.250	0.504	0.2462	0.3619	−0.5058	0.9981
Thinking time	Equal variances assumed	3.577	0.072	0.315	22	0.756	0.51483	1.63311	−2.87205	3.90170
	Equal variances not assumed			0.304	16.590	0.765	0.51483	1.69274	−3.06329	4.09294
Age	Equal variances assumed	2.099	0.161	0.217	22	0.831	1.126	5.198	−9.655	11.907
	Equal variances not assumed			0.224	21.086	0.825	1.126	5.033	−9.338	11.589

## Abbreviations

The following abbreviations are used in this manuscript:

A(r)	Agreement score
AR(r)	Agreement rate
GES	Gesture Elicitation Study
EOG	electrooculography
IoT	Internet-of-Things
